# Causal relationship between gut microbiota and risk of esophageal cancer: evidence from Mendelian randomization study

**DOI:** 10.18632/aging.205547

**Published:** 2024-02-15

**Authors:** Kui Wang, Jiawei Wang, Yuhua Chen, Huan Long, Wei Pan, Yunfei Liu, Ming-Yi Xu, Qiang Guo

**Affiliations:** 1 Department of Gastroenterology, The First People’s Hospital of Yunnan Province, The Affiliated Hospital of Kunming University of Science and Technology, Kunming 650032, Yunnan, China; 2 Medical School, Kunming University of Science and Technology, Kunming 650500, Yunnan Province, China; 3 Department of Critical Care Medicine, Jieyang Third People’s Hospital, Jieyang 515500, Guangdong Province, China; 4 Department of Gastroenterology, School of Medicine, Shanghai East Hospital, Tongji University, Shanghai 310115, China; 5 The First Clinical Medical College, Lanzhou University, Lanzhou 730000, Gansu Province, China; 6 Cardiology Department, Geriatrics Department, Foshan Women and Children Hospital, Foshan 528000, Guangdong, China; 7 University Munich, Munich D-81377, Germany

**Keywords:** esophageal cancer, genome-wide association study, comprehensive bidirectional Mendelian randomization, gut microbiota, causal association

## Abstract

Background: The causative implications remain ambiguous. Consequently, this study aims to evaluate the putative causal relationship between gut microbiota and Esophageal cancer (EC).

Methods: The genome-wide association study (GWAS) pertaining to the microbiome, derived from the MiBioGen consortium-which consolidates 18,340 samples across 24 population-based cohorts-was utilized as the exposure dataset. Employing the GWAS summary statistics specific to EC patients sourced from the GWAS Catalog and leveraging the two-sample Mendelian randomization (MR) methodology, the principal analytical method applied was the inverse variance weighted (IVW) technique. Cochran’s Q statistic was utilized to discern heterogeneity inherent in the data set. Subsequently, a reverse MR analysis was executed.

Results: Findings derived from the IVW technique elucidated that the Family Porphyromonadaceae (P = 0.048) and Genus Candidatus Soleaferrea (P = 0.048) function as deterrents against EC development. In contrast, the Genus Catenibacterium (P = 0.044), Genus Eubacterium coprostanoligenes group (P = 0.038), Genus Marvinbryantia (P = 0.049), Genus Ruminococcaceae UCG010 (P = 0.034), Genus Ruminococcus1 (P = 0.047), and Genus Sutterella (P = 0.012) emerged as prospective risk contributors for EC. To assess reverse causal effect, we used EC as the exposure and the gut microbiota as the outcome, and this analysis revealed associations between EC and seven different types of gut microbiota. The robustness of the MR findings was substantiated through comprehensive heterogeneity and pleiotropy evaluations.

Conclusions: This research identified certain microbial taxa as either protective or detrimental elements for EC, potentially offering valuable biomarkers for asymptomatic diagnosis and prospective therapeutic interventions for EC.

## INTRODUCTION

Esophageal carcinoma (EC) ranks as the seventh predominant malignancy globally, with roughly 604,100 documented incidences in 2020, constituting 3.1% of all cancerous diagnoses [1]. Additionally, this neoplastic disease accounted for an estimated 544,000 fatalities in the aforementioned year, signifying 5.5% of all oncology-associated mortalities, thereby placing it as the sixth leading etiology of cancer-induced death [2–4]. Globally, esophageal squamous cell carcinoma (ESCC) predominates as the principal histological subtype of esophageal malignancy, boasting an age-standardized incidence rate of 5.3 cases per 100,000 populaces [5, 6]. This incidence is quintuple that of esophageal adenocarcinoma (EAC), which has an age-standardized rate of 0.9 cases per 100,000 individuals [7, 8]. Individuals diagnosed with EC commonly manifest with advanced local progression and are frequently administered neoadjuvant chemoradiotherapy (CRT) or perioperative chemotherapy concomitant with surgical excision [9, 10]. Nonetheless, despite the successful culmination of conventional multidisciplinary treatment, numerous patients experience disease recurrence and ultimately succumb to the ailment [11, 12]. Factors that have been consistently recognized or associated with ESCC comprise tobacco smoking, alcohol ingestion, opium utilization, exposure to air contaminants, and specific dietary habits, notably a diminished intake of fruits and vegetables alongside an increased consumption of red meats and pickled products [13–15]. The complex etiological landscape of the disease hints at the existence of previously undetected elements influencing its pathogenesis. Notably, among these prospective contributors, the role of the gut microbiome is receiving amplified scholarly scrutiny [16]. The inception of the Human Microbiome Project, coupled with the accessibility of cost-effective high-throughput sequencing, has markedly transformed the direct exploration of the human microbiome’s impact on human health and pathophysiological conditions. The human microbiota has been suggested to be involved in tumorigenesis and the responsiveness to oncological treatments. The abundance, heterogeneity, and precise constitution of microbiota across various organs could potentially influence the pathogenesis of EC. Multiple research endeavors have posited that an imbalance within the gut microbial ecosystem, termed dysbiosis, could be implicated in the initiation and progression of esophageal neoplasia [17–23]. Individuals exhibiting reduced microbial richness in the esophagus and diminished salivary microbial diversity in China might be at an augmented risk of developing esophageal squamous dysplasia (a precursor to EC) and EC, correspondingly [24, 25]. An augmented abundance of *Clostridiales* and *Erysipelotrichales* in the gastric corpus may be implicated in the pathogenesis of esophageal squamous dysplasia and EC within the Iranian population [26]. Such discoveries have catalyzed inquiries into the nuanced interplay between the gut microbiome and EC. Regrettably, the determinations of the majority of contemporary observational research chiefly hinge upon the evaluation of the composition and alterations in gut microbiota derived from patients’ stool samples. Conventional observational research is constrained by intrinsic limitations, encompassing environmental confounders, selection biases, and the potential for reverse causality. While randomized controlled trials (RCTs) remain the benchmark for establishing causation, the vast multitude of gut microbial species combined with the extended latency from microbial dysbiosis to oncogenesis render RCTs challenging to implement within an authentic clinical context. In summation, the nexus between gut microbiota and Esophageal carcinoma continues to warrant further clarification.

Mendelian randomization (MR) serves as a robust approach for deducing causal relationships, employing genetic variants, such as Single Nucleotide Polymorphisms (SNP), as instrumental variables (IVs) [27]. Due to the stochastic allocation of alleles from parents to their descendants, coupled with their autonomous assortment and the postnatal stability of genotypes, MR is often likened to a quintessential RCT. The inherent benefits of MR, including the mitigation of confounding variables and the elimination of potential reverse causality, furnish a potent mechanism for deducing causal relationships in observational research [28–30]. Within this framework, MR presents an innovative methodology for investigating the putative causal nexus between gut microbiota and EC. In this investigation, two-sample MR analyses were conducted utilizing summary GWAS datasets to evaluate the potential causal linkage between gut microbiota and EC.

### Study design

**Figure 1:**
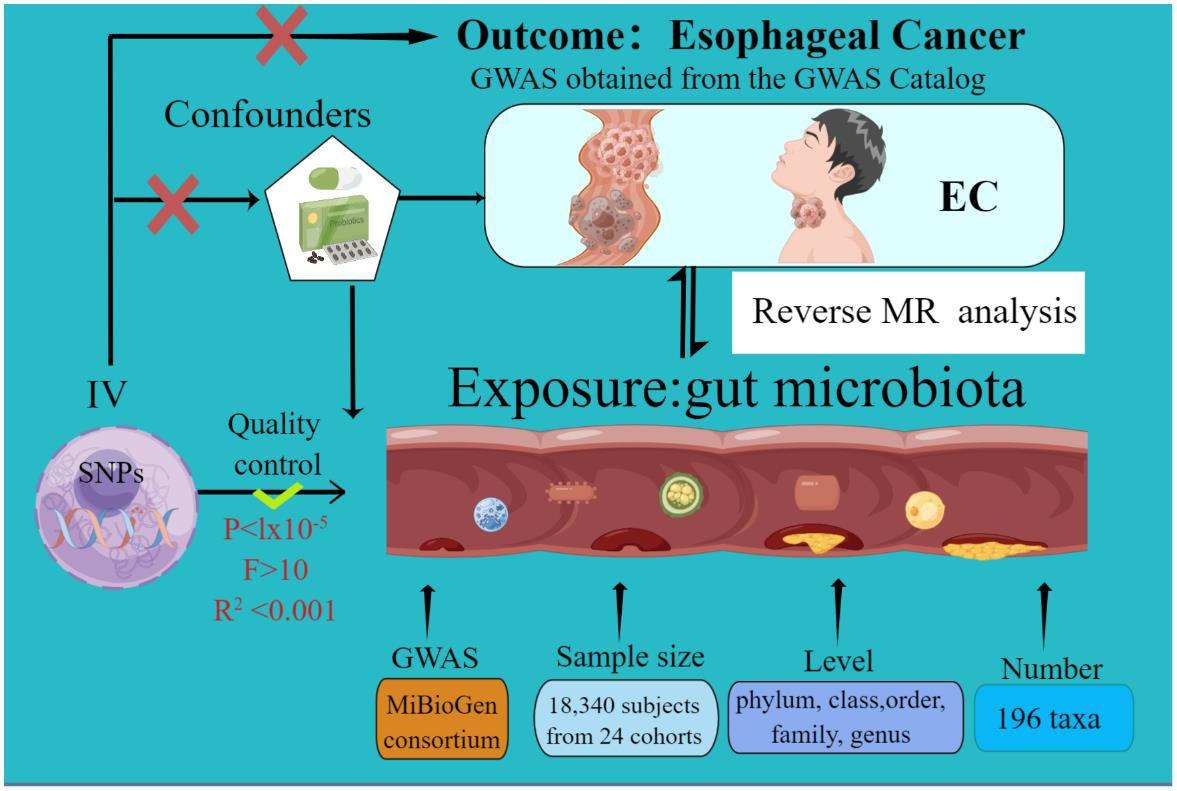
The study design of the present Mendelian randomization study of the associations of the gut microbiota and esophageal cancer risk.

The current MR study was executed and chronicled in accordance with the STROBE-MR guidelines. These guidelines are formulated to enhance the reporting rigor of MR research [31, 32]. The schematic representation of this study can be found in Figure 1. Credible outcomes hinge on the adherence to the following three foundational assumptions of Mendelian randomization analysis [33].

## MATERIALS AND METHODS

### Gut microbiome data sources

SNPs pertinent to the human gut microbiome composition were delineated as IVs from a GWAS dataset sourced from the international consortium MiBioGen. The MiBioGen Consortium, an international collaboration, is committed to advancing knowledge regarding the genetic architecture of gut microbiota. Data has been gathered from 24 population-based cohorts, cumulatively comprising 18,340 participants. In each respective cohort, the gut microbiota was analyzed using 16S rRNA sequencing methods, and the participants were genotyped utilizing whole-genome SNP arrays. The reference panel HRC 1.0 or 1.1 was utilized for the imputation of genotyping. Subsequently, factors including age, sex, technical variables, and genetic principal components were considered. An association analysis was subsequently conducted employing the Spearman correlation method. In this research, instrumental variables (IVs) were chosen from the genus to the phylum level of gut microbiota (GM) taxa. For an in-depth understanding, one is directed to consult the primary publications [34]. In the microbiota-GWAS, 122,110 variant sites spanning 211 taxa were identified. However, due to the presence of 12 unidentified genera and 3 unidentified families, a final total of 196 taxa were incorporated into the subsequent analysis.

### Esophageal cancer

The summary statistics pertaining to esophageal cancer were sourced from relevant studies listed in the GWAS Catalog (https://www.ebi.ac.uk/gwas/) under the identifier GCST90018841. This dataset comprised 998 cases of European descent and 475,308 controls, also of European ancestry [35].

### Selection of SNPs

To confirm the integrity and precision of the determinations regarding the causative association between the gut microbiome and the susceptibility to esophageal cancer, and to safeguard the data’ robustness and credibility, rigorous quality control procedures are imperative during the selection of the most suitable instrumental variables. The selection criteria of IVs were following: Prior literature was consulted to establish a more encompassing threshold (p < 1 × 10^–5^). Consequently, the threshold of p < 1 × 10^–5^ was employed due to the limited availability of suitable instrumental variables at p < 5 × 10^–8^ [36, 37]. Data from the 1000 Genomes project pertaining to European samples were utilized to calculate the linkage disequilibrium (LD) for SNPs with criteria set at R^2^ < 0.001 and a clumping distance of 10,000 kb. SNPs exhibiting the most significant P-values were subsequently retained for further analysis. In instances involving palindromic SNPs, allele frequencies were employed to deduce positive strand alleles. In the comparative analysis, alleles were cross-referenced with the Genome Reference Consortium Human Build 38, leading to the exclusion of ambiguous and redundant SNPs. In an effort to diminish heterogeneity and preclude pleiotropic influences, the MR Pleiotropy Residual Sum and Outlier (MR-PRESSO) approaches were utilized to pinpoint horizontal pleiotropic anomalies. We conducted a query on the PhenoScanner website to identify additional phenotypes linked to esophageal cancer-related SNPs. Subsequently, SNPs correlated with potential confounders, such as gastric cancer, biliary tract cancer, ovarian cancer, and so forth, were excluded from the analysis. Ultimately, we conducted an assessment of the strength of each individual IV via the F-statistics, denoted as F=β2 exposure/SE2 exposure. Additionally, an aggregate F-statistic was determined employing the subsequent formula: *F*=(*n*−*k*−1)*R*2/*k*(1−*R*2). Conventionally, an F-statistic greater than 10 was designated as the benchmark for robust IVs. Those not meeting this criterion were regarded as having a limited association with the exposure and were subsequently excluded [38].

### MR analysis

The statistical analysis was conducted using the R software (Version 4.2.2) complemented by the TwosampleMR package (Version 0.56). The MR study was employed to evaluate the potential causal relationships between 196 specific microbial taxa and esophageal cancer. Multiple methodologies were employed in our analysis, encompassing inverse variance weighting (IVW), weighted median, MR-Egger, weighted mode, and simple mode techniques. Given that the IVW approach yields the most accurate causal estimations, it was designated as the principal analytical technique for this MR investigation [39]. The MR-Egger method discerns horizontal pleiotropy by evaluating the intercept. An intercept yielding a p-value below 0.05 suggests the existence of pleiotropy [40]. The heterogeneity was assessed using Cochran’s Q test. The MR-PRESSO global test was employed to mitigate horizontal pleiotropy by identifying and excluding definitive outliers [41–43]. Additionally, to pinpoint SNPs that might exhibit heterogeneity, a “leave-one-out” procedure was executed, wherein each SNP was systematically excluded in turn.

### Reverse MR analysis

To explore the putative causal link between esophageal cancer and diverse bacteria, we undertook a reverse MR study. SNPs meeting the locus-specific significance criterion (P < 1.0×10^–5^) were identified as potential IVs. In this framework, esophageal cancer was designated as the exposure, while the gut microbiota composition was considered the outcome. Instrumental variables for this investigation were constituted by SNPs associated with esophageal cancer.

### Ethical approval

This research utilized publicly accessible data. Each individual study within the GWAS was sanctioned by its respective Institutional Review Board, and informed consent was secured either directly from the participants or through a designated caregiver, legal guardian, or equivalent representative.

### Availability of data and materials

https://mrcieu.github.io/TwoSampleMR/, https://github.com/rondolab/MR-PRESSO.

### Consent for publication

Consensus among all authors was achieved regarding the manuscript.

## RESULTS

### Genetic instruments for gut microbiome

In the research undertaken, 196 bacterial characteristics encompassing five hierarchical biological levels (namely, phylum, class, order, family, and genus) were analyzed. Comprehensive data pertaining to the concluding SNPs associated with each bacterial trait can be found in Supplementary Tables 1, 2. F values exceeding 10 suggest an absence of any weak instrument bias. For all MR results, we conducted comprehensive sensitivity analyses to investigate potential heterogeneity, as evidenced by Cochran’s Q statistic, and to assess pleiotropic influences using both MR-Egger regression and MR-PRESSO techniques.

### Causal effect of gut microbiota on esophageal cancer

**Figure 2:**
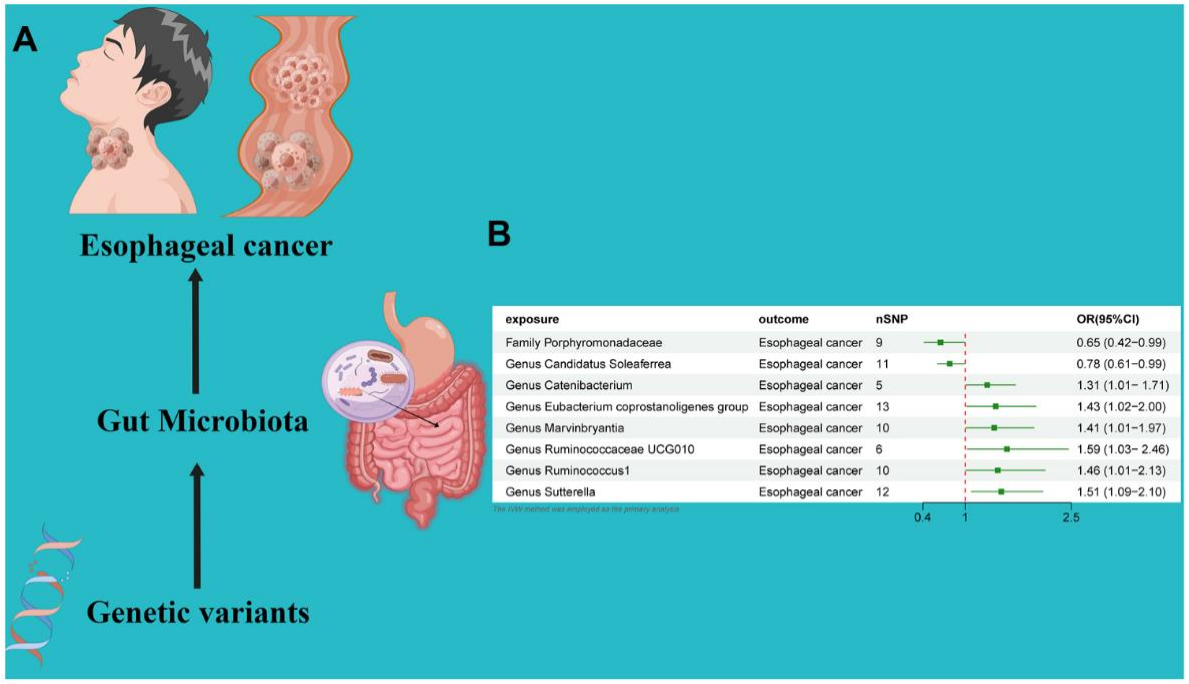
(**A**) Causal effect of gut microbiota with Esophageal cancer Schematic representation of the MR analysis results. (**B**) Forest plot of the MR analysis results.

**Figure 3:**
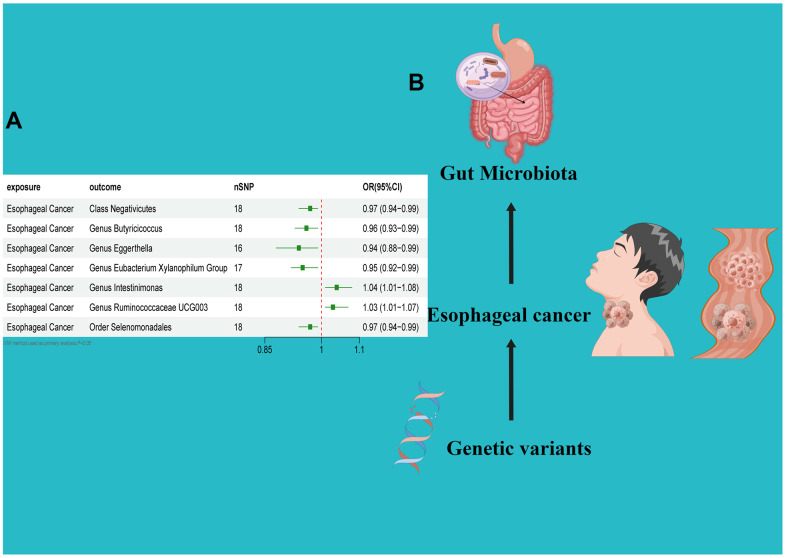
(**A**) Forest plot of the Reverse MR analysis results. (**B**) Causal effect of Esophageal cancer with gut microbiota Schematic representation of the Reverse MR analysis results. OR odds ratio, CI confidence interval, IVW inverse variance weighted method, Significant threshold was set at p-value <0.05 for the Inverse Variance Weighted method (IVW).

An MR analysis was performed to ascertain the potential causal linkage between gut microbiota and esophageal cancer. Detailed findings are presented in Table 1. Through the application of the IVW analytical approach, we determined that the *Family Streptococcaceae* (OR 0.65, 95% CI 0.42–0.99, *P* = 0.048) and Genus Candidatus Soleaferrea(OR 0.78, 95% CI 0.61–0.99, *P* = 0.048) demonstrated an inverse association with susceptibility to esophageal cancer. In contrast, positive correlations with esophageal cancer risk were evident for the *Genus Catenibacterium* (OR=1.31, 95% CI:1.01–1.71, *P*=0.044), *Genus Eubacterium coprostanoligenes* (OR=1.43, 95% CI:1.02–2.00, *P*=0.038), *Genus Marvinbryantia* (OR=1.41, 95% CI:1.01–1.97, *P*=0.049), *Genus Ruminococcaceae UCG010* (OR=1.59, 95% CI:1.03–2.46, *P*=0.034), *Genus Ruminococcus1* (OR=1.46, 95% CI:1.01–2.13, *P*=0.047), and *Genus Sutterella* (OR=1.51, 95% CI:1.09–2.10, *P*=0.012). (Figure 2) Utilizing both the IVW test and MR-Egger, the outcomes of Cochran’s Q test revealed an absence of notable heterogeneity between the gut microbiome and esophageal cancer. The MR-Egger regression analysis provided no indications of horizontal pleiotropy. Furthermore, the MR-PRESSO evaluation identified no significant outliers, and the leave-one-out analysis corroborated the robustness of the data (Table 1 and Supplementary Table 2 and Figures 2, 3).

**Table 1:** Summary results of MR (target gut microbiome on esophageal cancer).

**Taxa**	**Exposure**	**Outcome**	**Nsnp**	**Methods**	**Beta**	**SE**	**OR (95%CI)**	***P*-value**	**Heterogeneity**		**Horizontal pleiotrop**	
									**Cochran’s Q**	**P-value**	**Egger intercept P**	**MR-PRESSO P**
Family	Porphyromonadaceae	Esophageal cancer	9	Inverse variance weighted	-0.432	0.219	0.65 (0.42-0.99)	0.048	6.708	0.568	0.279	0.6
Genus	Candidatus Soleaferrea	Esophageal cancer	11	Inverse variance weighted	-0.248	0.125	0.78 (0.61-0.99)	0.048	10.878	0.367	0.594	0.38
Genus	Catenibacterium	Esophageal cancer	5	Inverse variance weighted	0.196	0.134	1.31 (1.01- 1.71)	0.044	1.795	0.773	0.924	0.78
Genus	Eubacterium coprostanoligenes	Esophageal cancer	13	Inverse variance weighted	0.355	0.171	1.43 (1.02-2.00)	0.038	9.402	0.668	0.718	0.68
Genus	Marvinbryantia	Esophageal cancer	10	Inverse variance weighted	0.340	0.173	1.41 (1.01-1.97)	0.049	3.315	0.950	0.813	0.97
Genus	Ruminococcaceae UCG010	Esophageal cancer	6	Inverse variance weighted	0.466	0.220	1.59 (1.03- 2.46)	0.034	2.971	0.704	0.863	0.69
Genus	Ruminococcus1	Esophageal cancer	10	Inverse variance weighted	0.381	0.192	1.46 (1.01-2.13)	0.047	7.057	0.631	0.090	0.62
Genus	Sutterella	Esophageal cancer	12	Inverse variance weighted	0.414	0.166	1.51 (1.09-2.10)	0.012	6.918	0.805	0.116	0.93

### Causal effect of esophageal cancer on gut microbiota

**Figure 4:**
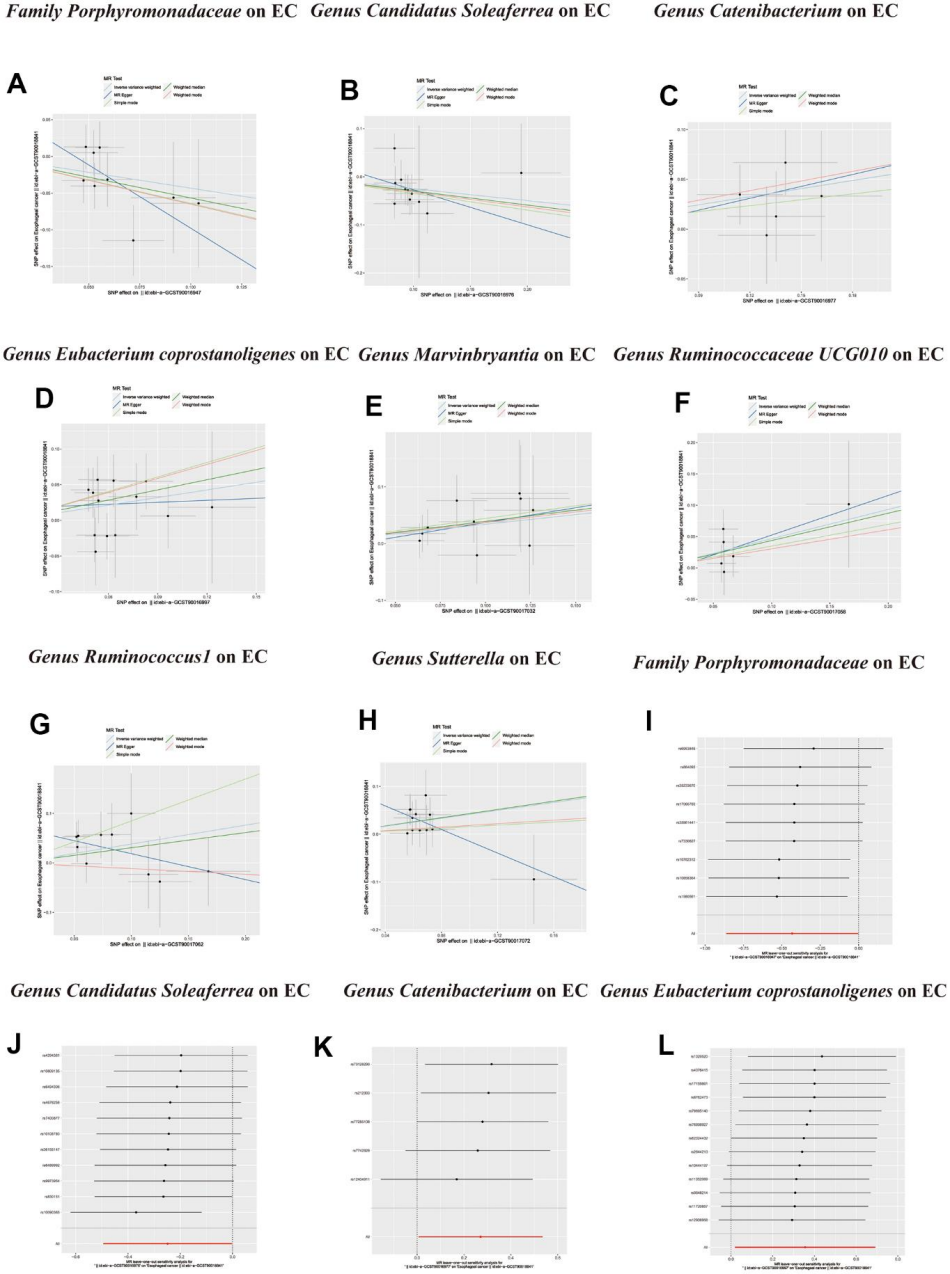
**Scatter plots of significant causality of the GM and esophageal cancer.** (**A**–**H**) Scatter plot of the effect size and 95% confidence interval of each SNP on GM and Esophageal cancer risk. The horizontal axis reflects genetic effect of each SNP on GM. The vertical axis represents the genetic effect of each SNP on Esophageal cancer risk. Leave-one-out analysis for the impact of individual SNPs on the association between GM and Esophageal cancer risk. (**I**–**L**) By leaving out exactly one SNP, it demonstrates how each individual SNP influences the overall estimate.

**Figure 5:**
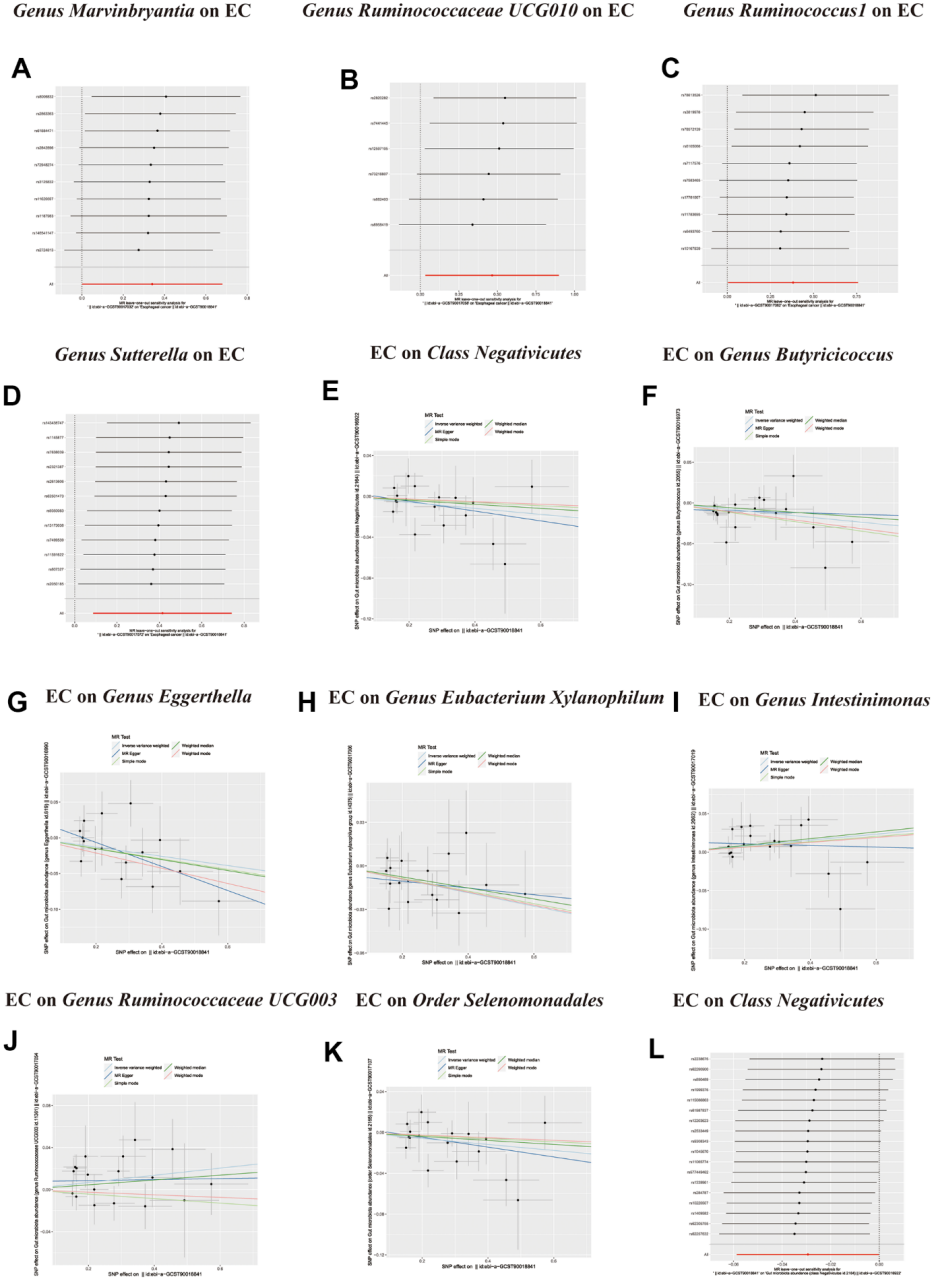
(**A**–**D**) Leave-one-out analysis for the impact of individual SNPs on the association between GM and Esophageal cancer risk. (**E**–**K**) In reverse MR analysis, The scatter plots for association between Esophageal cancer and gut microbiota. (**L**) In reverse MR analysis, Plots for “leave-one-out” analysis for causal effect of Esophageal cancer on gut microbiota risk.

**Figure 6:**
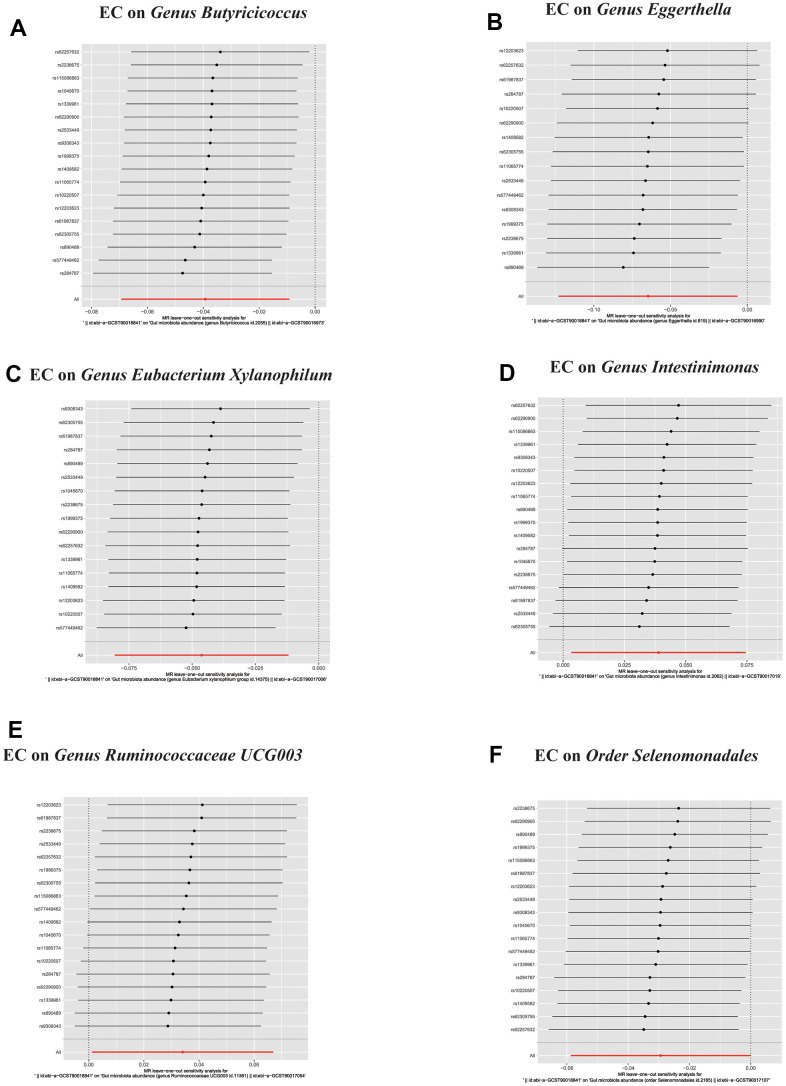
In reverse MR analysis, (**A**–**F**) Plots for “leave-one-out” analysis for causal effect of Esophageal cancer on gut microbiota risk.

In the reverse direction MR analysis, we used Esophageal cancer as the exposure and gut microbiota as the outcome to assess any reverse causation effects. After analysis using the MR method, Esophageal cancer had a causal effect on one *Class*, one *Order* and five *Genera*. Through IVW, the *Class Negativicutes* (OR=0.97, 95% CI:0.94–0.99, *P*=0.048), *Order* *Selenomonadales* (OR=0.97, 95% CI:0.94–0.99, *P*=0.048), *Genus Butyricicoccus* (OR=0.96, 95% CI:0.93–0.99, *P*=0.010), *Genus Eggerthella* (OR=0.94, 95% CI:0.88–0.99, *P*=0.029) and *Genus Eubacterium Xylanophilum Group* (OR=0.95, 95% CI:0.92–0.99, *P*=0.008) were down-regulated after the onset of Esophageal cancer. The *Genus Intestinimonas* (OR=1.04, 95% CI:1.01–1.08, *P*=0.032) and *Genus Ruminococcaceae UCG003* (OR=1.03, 95% CI:1.01–1.07, *P*=0.042) were up-regulated after the onset of Esophageal cancer. Among the IVs, we detected neither weak instrument bias nor notable heterogeneity statistics. Additionally, no evidence of horizontal pleiotropy was observed between the IVs and the gut microbiome. The MR-PRESSO assessment revealed an absence of significant outliers. Moreover, the robustness of the data was corroborated through the leave-one-out analysis (Table 2 and Supplementary Tables 3, 4 and Figures 4–6).

**Table 2:** Summary results of bidirectional MR (esophageal cancer on target gut microbiome).

**Exposure**	**Taxa**	**Outcome**	**Nsnp**	**Methods**	**Beta**	**SE**	**OR (95%CI)**	***P*-value**	**Heterogeneity**		**Horizontal pleiotrop**	
									**Cochran’s Q**	**P-value**	**Egger intercept P**	**MR-PRESSO P**
Esophageal cancer	Class	Negativicutes	18	Inverse variance weighted	-0.029	0.014	0.97 (0.94-0.99)	0.048	15.619	0.550	0.611	0.604
Esophageal cancer	Order	Selenomonadales	18	Inverse variance weighted	-0.029	0.014	0.97 (0.94-0.99)	0.048	15.619	0.550	0.611	0.38
Esophageal cancer	Genus	Butyricicoccus	18	Inverse variance weighted	-0.039	0.015	0.96 (0.93-0.99)	0.010	14.124	0.658	0.464	0.78
Esophageal cancer	Genus	Eggerthella	16	Inverse variance weighted	-0.064	0.029	0.94 (0.88-0.99)	0.029	17.484	0.290	0.162	0.68
Esophageal cancer	Genus	Eubacterium Xylanophilum Group	17	Inverse variance weighted	-0.046	0.017	0.95 (0.92-0.99)	0.008	9.596	0.886	0.599	0.97
Esophageal cancer	Genus	Intestinimonas	18	Inverse variance weighted	0.038	0.018	1.04 (1.01-1.08)	0.032	15.844	0.534	0.270	0.69
Esophageal cancer	Genus	Ruminococcaceae UCG003	18	Inverse variance weighted	0.034	0.016	1.03 (1.01-1.07)	0.042	15.184	0.582	0.481	0.62

## DISCUSSION

Our research is the inaugural comprehensive bidirectional MR analysis investigating the potential causal association between gut microbiota and esophageal cancer. Utilizing GWAS summary data, we corroborated a linkage between esophageal cancer and the gut microbiome, and our findings align congruently with existing scholarly literature. Our study discerned a bidirectional interaction between esophageal cancer and the gut microbiome. We identified specific risk factors, including the *Genera Catenibacterium*, *Eubacterium coprostanoligenes group*, *Marvinbryantia, Ruminococcaceae UCG010, Ruminococcus1*, and *Sutterella*. In contrast, protective factors, such as the *Family Porphyromonadaceae* and the genus *Candidatus Soleaferrea*, were observed to be linked with esophageal cancer within the gut microbiome. The emergence of esophageal cancer manifested alterations in the gut microbiome composition. For instance, an elevation in the concentrations of the *genera Intestinimonas* and *Ruminococcaceae UCG003* was noted, while there was a discernible decrease in the relative abundance of the *Class Negativicutes* and the genera *Butyricicoccus, Eggerthella, Eubacterium Xylanophilum Group*, coupled with the *order Selenomonadales.* EC represents a virulent neoplasia characterized by a bleak prognostic outcome. Microbiome research represents an emergent and swiftly progressing domain within oncological studies pertaining to humans [44, 45]. In recent academic epochs, novel research has enriched our understanding of the association between shifts in the gut microbiota and the onset of esophageal carcinogenesis [46]. A myriad of determinants, encompassing dietary practices, along with antacid and antibiotic utilization, have been demonstrably linked to modulations in the esophageal microbiome. Inversely, the amplification and heterogeneity of the esophageal microbiome can reciprocally affect its functional dynamics [47–50]. Multiple academic inquiries have discerned a pronounced presence of *Campylobacter* in BE patients, an observation absent in normative subjects [51–53]. BE markedly elevates the susceptibility to EAC development, with a risk amplification up to 30-fold relative to individuals devoid of BE [54]. Shao et al. delineated a diminished microbial diversity in ESCC specimens in comparison to non-neoplastic tissues, as ascertained through 16S rDNA sequencing. Notably, they observed a pronounced augmentation in the prevalence of Fusobacterium and a concomitant reduction in Streptococcus presence [55]. Li et al. elucidated a marked diminution in the prevalence of Streptococcus concomitant with an enhanced presence of Neisseria and Porphyromonas in the course of ESCC progression [56]. Such incongruities might stem from variations in dietary patterns, geographical locales, and disparities in the patient cohorts encompassed by the individual studies. Nonetheless, these investigations, in aggregate, underscore the prevailing perturbations in the microbial homeostasis of the esophagus among ESCC patients [57–62].

Alterations in both the composition and the prevalence of microbiota within the esophagus may contribute to the pathogenesis of EC through various mechanisms [63, 64]. The dysregulation of interactions between the esophageal microbiota and immune cells has been implicated in the modulation of multiple signaling pathways, which are known to play a significant role in the etiology of EC [65]. Dysfunctional activation of the Wnt/β-catenin signaling pathway has been associated with both the oncogenesis and therapeutic resistance observed in EC [66]. Fusobacterium nucleatum facilitates the progression and chemotherapeutic resistance of esophageal squamous cell carcinoma by augmenting the release of senescence-associated secretory phenotype induced by chemotherapy, through the activation of the DNA damage response pathway.

Our research possesses distinct strengths. To the best of our understanding, this constitutes the inaugural MR analysis probing the potential causal association between the gut microbiome and esophageal cancer. The MR methodology intrinsically reduces susceptibilities to disruptions from lingering confounders, potentially presenting a more steadfast paradigm in comparison to traditional observational approaches. However, in light of the prevailing uncertainties surrounding the exact biological functions of certain genetic variants, the possible effects of horizontal pleiotropy cannot be fully negated. As such, it is imperative to interpret our results with prudence. Moreover, we conducted an in-depth examination of the causal relationships of individual taxa with esophageal cancer, encompassing hierarchical levels from phyla to genera. This exploration offers innovative insights into the intricate mechanisms associated with the gut microbiome and suggests potential therapeutic strategies centered on microbiome modulation. However, inherent constraints persist in our analysis. It is important to note that, despite our examination of prevalent gut microbiota, the vast and diverse nature of the gut microbiome means that our dataset may yet have its limitations. Additionally, our research focused exclusively on European populations and did not differentiate based on gender. Consequently, extrapolating our conclusions to broader populations requires judicious consideration. Notwithstanding its constraints, this study furnishes salient insights into the causative associations between gut microbiota and esophageal cancer, thus establishing a foundational premise for ensuing explorations into the intrinsic molecular dynamics.

## CONCLUSIONS

In this MR analysis, we present an inaugural thorough examination of the causal relationships between gut microbiota and esophageal cancer. Our results offer innovative understandings concerning the prophylaxis, disease progression, and therapeutic strategies for esophageal cancer by focusing on distinct bacterial taxa. Subsequent research is imperative to elucidate the precise mechanistic interplay between enteric microbiota and esophageal cancer associations. Nonetheless, additional investigations are requisite to ascertain the underlying mechanism delineating the association between gut microbiota and esophageal cancer.

## Supplementary Material

Supplementary Table 1

Supplementary Table 2

Supplementary Table 3

Supplementary Table 4

## References

[1] Holmberg D, Santoni G, von Euler-Chelpin M, Färkkilä M, Kauppila JH, Maret-Ouda J, Ness-Jensen E, Lagergren J. Non-erosive gastro-oesophageal reflux disease and incidence of oesophageal adenocarcinoma in three Nordic countries: population based cohort study. BMJ. 2023; 382:e076017. 10.1136/bmj-2023-076017 37704252 PMC10496574

[2] Sung H, Ferlay J, Siegel RL, Laversanne M, Soerjomataram I, Jemal A, Bray F. Global Cancer Statistics 2020: GLOBOCAN Estimates of Incidence and Mortality Worldwide for 36 Cancers in 185 Countries. CA Cancer J Clin. 2021; 71:209–49. 10.3322/caac.21660 33538338

[3] Sharma P. Barrett Esophagus: A Review. JAMA. 2022; 328:663–71. 10.1001/jama.2022.13298 35972481

[4] Coleman HG, Xie SH, Lagergren J. The Epidemiology of Esophageal Adenocarcinoma. Gastroenterology. 2018; 154:390–405. 10.1053/j.gastro.2017.07.046 28780073

[5] GBD 2017 Oesophageal Cancer Collaborators. The global, regional, and national burden of oesophageal cancer and its attributable risk factors in 195 countries and territories, 1990-2017: a systematic analysis for the Global Burden of Disease Study 2017. Lancet Gastroenterol Hepatol. 2020; 5:582–97. 10.1016/S2468-1253(20)30007-8 32246941 PMC7232026

[6] Arnold M, Soerjomataram I, Ferlay J, Forman D. Global incidence of oesophageal cancer by histological subtype in 2012. Gut. 2015; 64:381–7. 10.1136/gutjnl-2014-308124 25320104

[7] He H, Chen N, Hou Y, Wang Z, Zhang Y, Zhang G, Fu J. Trends in the incidence and survival of patients with esophageal cancer: A SEER database analysis. Thorac Cancer. 2020; 11:1121–8. 10.1111/1759-7714.13311 32154652 PMC7180574

[8] Morgan E, Soerjomataram I, Rumgay H, Coleman HG, Thrift AP, Vignat J, Laversanne M, Ferlay J, Arnold M. The Global Landscape of Esophageal Squamous Cell Carcinoma and Esophageal Adenocarcinoma Incidence and Mortality in 2020 and Projections to 2040: New Estimates From GLOBOCAN 2020. Gastroenterology. 2022; 163:649–58.e2. 10.1053/j.gastro.2022.05.054 35671803

[9] Shah MA, Hofstetter WL, Kennedy EB, and Locally Advanced Esophageal Carcinoma Guideline Expert Panel. Immunotherapy in Patients With Locally Advanced Esophageal Carcinoma: ASCO Treatment of Locally Advanced Esophageal Carcinoma Guideline Rapid Recommendation Update. J Clin Oncol. 2021; 39:3182–4. 10.1200/JCO.21.01831 34406872

[10] Arnold M, Ferlay J, van Berge Henegouwen MI, Soerjomataram I. Global burden of oesophageal and gastric cancer by histology and subsite in 2018. Gut. 2020; 69:1564–71. 10.1136/gutjnl-2020-321600 32606208

[11] Shah MA, Altorki N, Patel P, Harrison S, Bass A, Abrams JA. Improving outcomes in patients with oesophageal cancer. Nat Rev Clin Oncol. 2023; 20:390–407. 10.1038/s41571-023-00757-y 37085570

[12] He S, Xu J, Liu X, Zhen Y. Advances and challenges in the treatment of esophageal cancer. Acta Pharm Sin B. 2021; 11:3379–92. 10.1016/j.apsb.2021.03.008 34900524 PMC8642427

[13] Zang Z, Liu Y, Wang J, Liu Y, Zhang S, Zhang Y, Zhang L, Zhao D, Liu F, Chao L, Wang X, Zhang C, Song G, et al. Dietary patterns and severity of symptom with the risk of esophageal squamous cell carcinoma and its histological precursor lesions in China: a multicenter cross-sectional latent class analysis. BMC Cancer. 2022; 22:95. 10.1186/s12885-022-09206-y 35062901 PMC8783423

[14] Abnet CC, Arnold M, Wei WQ. Epidemiology of Esophageal Squamous Cell Carcinoma. Gastroenterology. 2018; 154:360–73. 10.1053/j.gastro.2017.08.023 28823862 PMC5836473

[15] Yang J, Liu X, Cao S, Dong X, Rao S, Cai K. Understanding Esophageal Cancer: The Challenges and Opportunities for the Next Decade. Front Oncol. 2020; 10:1727. 10.3389/fonc.2020.01727 33014854 PMC7511760

[16] Wu H, Leng X, Liu Q, Mao T, Jiang T, Liu Y, Li F, Cao C, Fan J, Chen L, Chen Y, Yao Q, Lu S, et al. Intratumoral Composition Regulates Microbiota Chemoimmunotherapy Response in Esophageal Squamous Cell Carcinoma. Cancer Res. 2023; 83:3131–44. 10.1158/0008-5472.CAN-22-2593 37433041

[17] Yamamura K, Baba Y, Nakagawa S, Mima K, Miyake K, Nakamura K, Sawayama H, Kinoshita K, Ishimoto T, Iwatsuki M, Sakamoto Y, Yamashita Y, Yoshida N, et al. Human Microbiome Fusobacterium Nucleatum in Esophageal Cancer Tissue Is Associated with Prognosis. Clin Cancer Res. 2016; 22:5574–81. 10.1158/1078-0432.CCR-16-1786 27769987

[18] Sugimoto T, Atobe S, Kado Y, Takahashi A, Motoori M, Sugimura K, Miyata H, Yano M, Tanaka K, Doki Y, Shiraishi O, Yasuda T, Asahara T. Gut microbiota associated with the mitigation effect of synbiotics on adverse events of neoadjuvant chemotherapy in patients with esophageal cancer: A retrospective exploratory study. J Med Microbiol. 2023; 72. 10.1099/jmm.0.001723 37367942

[19] Baba Y, Hara Y, Toihata T, Kosumi K, Iwatsuki M, Iwagami S, Miyamoto Y, Yoshida N, Komohara Y, Baba H. Relationship between gut microbiome Fusobacterium nucleatum and LINE-1 methylation level in esophageal cancer. Esophagus. 2023; 20:704–12. 10.1007/s10388-023-01009-9 37173453

[20] Sasaki T, Matsumoto Y, Murakami K, Endo S, Toyozumi T, Otsuka R, Kinoshita K, Hu J, Iida S, Morishita H, Nishioka Y, Nakano A, Uesato M, Matsubara H. Gut microbiome can predict chemoradiotherapy efficacy in patients with esophageal squamous cell carcinoma. Esophagus. 2023; 20:691–703. 10.1007/s10388-023-01004-0 37086309

[21] Shi Z, Li H, Song W, Zhou Z, Li Z, Zhang M. Emerging roles of the gut microbiota in cancer immunotherapy. Front Immunol. 2023; 14:1139821. 10.3389/fimmu.2023.1139821 36911704 PMC9992551

[22] Muszyński D, Kudra A, Sobocki BK, Folwarski M, Vitale E, Filetti V, Dudzic W, Kaźmierczak-Siedlecka K, Połom K. Esophageal cancer and bacterial part of gut microbiota - A multidisciplinary point of view. Front Cell Infect Microbiol. 2022; 12:1057668. 10.3389/fcimb.2022.1057668 36467733 PMC9709273

[23] Zhang L, Chai D, Chen C, Li C, Qiu Z, Kuang T, Parveena M, Dong K, Yu J, Deng W, Wang W. Mycobiota and C-Type Lectin Receptors in Cancers: Know thy Neighbors. Front Microbiol. 2022; 13:946995. 10.3389/fmicb.2022.946995 35910636 PMC9326027

[24] Yu G, Dye BA, Gail MH, Shi J, Klepac-Ceraj V, Paster BJ, Wang GQ, Wei WQ, Fan JH, Qiao YL, Dawsey SM, Freedman ND, Abnet CC. The association between the upper digestive tract microbiota by HOMIM and oral health in a population-based study in Linxian, China. BMC Public Health. 2014; 14:1110. 10.1186/1471-2458-14-1110 25348940 PMC4223728

[25] Killcoyne S, Fitzgerald RC. Evolution and progression of Barrett’s oesophagus to oesophageal cancer. Nat Rev Cancer. 2021; 21:731–41. 10.1038/s41568-021-00400-x 34545238

[26] Nasrollahzadeh D, Malekzadeh R, Ploner A, Shakeri R, Sotoudeh M, Fahimi S, Nasseri-Moghaddam S, Kamangar F, Abnet CC, Winckler B, Islami F, Boffetta P, Brennan P, et al. Variations of gastric corpus microbiota are associated with early esophageal squamous cell carcinoma and squamous dysplasia. Sci Rep. 2015; 5:8820. 10.1038/srep08820 25743945 PMC4351546

[27] Zhong MM, Xie JH, Feng Y, Zhang SH, Xia JN, Tan L, Chen NX, Su XL, Zhang Q, Feng YZ, Guo Y. Causal effects of the gut microbiome on COVID-19 susceptibility and severity: a two-sample Mendelian randomization study. Front Immunol. 2023; 14:1173974. 10.3389/fimmu.2023.1173974 37720222 PMC10502427

[28] Tin A, Köttgen A. Mendelian Randomization Analysis as a Tool to Gain Insights into Causes of Diseases: A Primer. J Am Soc Nephrol. 2021; 32:2400–7. 10.1681/ASN.2020121760 34135084 PMC8722812

[29] Swanson SA, Labrecque J, Hernán MA. Causal null hypotheses of sustained treatment strategies: What can be tested with an instrumental variable? Eur J Epidemiol. 2018; 33:723–8. 10.1007/s10654-018-0396-6 29721747 PMC6061140

[30] Wang K, Qin X, Ran T, Pan Y, Hong Y, Wang J, Zhang X, Shen X, Liu C, Lu X, Chen Y, Bai Y, Zhang Y, et al. Causal link between gut microbiota and four types of pancreatitis: a genetic association and bidirectional Mendelian randomization study. Front Microbiol. 2023; 14:1290202. 10.3389/fmicb.2023.1290202 38075894 PMC10702359

[31] Skrivankova VW, Richmond RC, Woolf BAR, Davies NM, Swanson SA, VanderWeele TJ, Timpson NJ, Higgins JPT, Dimou N, Langenberg C, Loder EW, Golub RM, Egger M, et al. Strengthening the reporting of observational studies in epidemiology using mendelian randomisation (STROBE-MR): explanation and elaboration. BMJ. 2021; 375:n2233. 10.1136/bmj.n2233 34702754 PMC8546498

[32] Skrivankova VW, Richmond RC, Woolf BAR, Yarmolinsky J, Davies NM, Swanson SA, VanderWeele TJT, Higgins JP, Timpson NJ, Dimou N, Langenberg C, Golub RM, Loder EW, et al. Strengthening the Reporting of Observational Studies in Epidemiology Using Mendelian Randomization: The STROBE-MR Statement. JAMA. 2021; 326:1614–21. 10.1001/jama.2021.18236 34698778

[33] de Leeuw C, Savage J, Bucur IG, Heskes T, Posthuma D. Understanding the assumptions underlying Mendelian randomization. Eur J Hum Genet. 2022; 30:653–60. 10.1038/s41431-022-01038-5 35082398 PMC9177700

[34] Kurilshikov A, Medina-Gomez C, Bacigalupe R, Radjabzadeh D, Wang J, Demirkan A, Le Roy CI, Raygoza Garay JA, Finnicum CT, Liu X, Zhernakova DV, Bonder MJ, Hansen TH, et al. Large-scale association analyses identify host factors influencing human gut microbiome composition. Nat Genet. 2021; 53:156–65. 10.1038/s41588-020-00763-1 33462485 PMC8515199

[35] Sakaue S, Kanai M, Tanigawa Y, Karjalainen J, Kurki M, Koshiba S, Narita A, Konuma T, Yamamoto K, Akiyama M, Ishigaki K, Suzuki A, Suzuki K, et al., and FinnGen. A cross-population atlas of genetic associations for 220 human phenotypes. Nat Genet. 2021; 53:1415–24. 10.1038/s41588-021-00931-x 34594039 PMC12208603

[36] Li Z, Zhu G, Lei X, Tang L, Kong G, Shen M, Zhang L, Song L. Genetic support of the causal association between gut microbiome and COVID-19: a bidirectional Mendelian randomization study. Front Immunol. 2023; 14:1217615. 10.3389/fimmu.2023.1217615 37483615 PMC10360131

[37] Zhang L, Zi L, Kuang T, Wang K, Qiu Z, Wu Z, Liu L, Liu R, Wang P, Wang W. Investigating causal associations among gut microbiota, metabolites, and liver diseases: a Mendelian randomization study. Front Endocrinol (Lausanne). 2023; 14:1159148. 10.3389/fendo.2023.1159148 37476494 PMC10354516

[38] Pierce BL, Ahsan H, Vanderweele TJ. Power and instrument strength requirements for Mendelian randomization studies using multiple genetic variants. Int J Epidemiol. 2011; 40:740–52. 10.1093/ije/dyq151 20813862 PMC3147064

[39] Gill D, Burgess S. The evolution of mendelian randomization for investigating drug effects. PLoS Med. 2022; 19:e1003898. 10.1371/journal.pmed.1003898 35113864 PMC8812877

[40] Bowden J, Davey Smith G, Burgess S. Mendelian randomization with invalid instruments: effect estimation and bias detection through Egger regression. Int J Epidemiol. 2015; 44:512–25. 10.1093/ije/dyv080 26050253 PMC4469799

[41] Verbanck M, Chen CY, Neale B, Do R. Detection of widespread horizontal pleiotropy in causal relationships inferred from Mendelian randomization between complex traits and diseases. Nat Genet. 2018; 50:693–8. 10.1038/s41588-018-0099-7 29686387 PMC6083837

[42] Cho Y, Haycock PC, Sanderson E, Gaunt TR, Zheng J, Morris AP, Davey Smith G, Hemani G. Exploiting horizontal pleiotropy to search for causal pathways within a Mendelian randomization framework. Nat Commun. 2020; 11:1010. 10.1038/s41467-020-14452-4 32081875 PMC7035387

[43] Sanderson E, Glymour MM, Holmes MV, Kang H, Morrison J, Munafò MR, Palmer T, Schooling CM, Wallace C, Zhao Q, Smith GD. Mendelian randomization. Nat Rev Methods Primers. 2022; 2:6. 10.1038/s43586-021-00092-5 37325194 PMC7614635

[44] Liu Y, Baba Y, Ishimoto T, Iwatsuki M, Hiyoshi Y, Miyamoto Y, Yoshida N, Wu R, Baba H. Progress in characterizing the linkage between Fusobacterium nucleatum and gastrointestinal cancer. J Gastroenterol. 2019; 54:33–41. 10.1007/s00535-018-1512-9 30244399

[45] Baba Y, Iwatsuki M, Yoshida N, Watanabe M, Baba H. Review of the gut microbiome and esophageal cancer: Pathogenesis and potential clinical implications. Ann Gastroenterol Surg. 2017; 1:99–104. 10.1002/ags3.12014 29863142 PMC5881342

[46] Deshpande NP, Riordan SM, Castaño-Rodríguez N, Wilkins MR, Kaakoush NO. Signatures within the esophageal microbiome are associated with host genetics, age, and disease. Microbiome. 2018; 6:227. 10.1186/s40168-018-0611-4 30558669 PMC6297961

[47] Zhang JW, Zhang D, Yin HS, Zhang H, Hong KQ, Yuan JP, Yu BP. Fusobacterium nucleatum promotes esophageal squamous cell carcinoma progression and chemoresistance by enhancing the secretion of chemotherapy-induced senescence-associated secretory phenotype via activation of DNA damage response pathway. Gut Microbes. 2023; 15:2197836. 10.1080/19490976.2023.2197836 37017266 PMC10078122

[48] Lei J, Xu F, Deng C, Nie X, Zhong L, Wu Z, Li J, Wu X, He S, Chen Y. Fusobacterium nucleatum promotes the early occurrence of esophageal cancer through upregulation of IL-32/PRTN3 expression. Cancer Sci. 2023; 114:2414–28. 10.1111/cas.15787 36919771 PMC10236610

[49] Qiao H, Li H, Wen X, Tan X, Yang C, Liu N. Multi-Omics Integration Reveals the Crucial Role of Fusobacterium in the Inflammatory Immune Microenvironment in Head and Neck Squamous Cell Carcinoma. Microbiol Spectr. 2022; 10:e0106822. 10.1128/spectrum.01068-22 35862975 PMC9431649

[50] Cheung MK, Yue GGL, Tsui KY, Gomes AJ, Kwan HS, Chiu PWY, Lau CBS. Discovery of an interplay between the gut microbiota and esophageal squamous cell carcinoma in mice. Am J Cancer Res. 2020; 10:2409–27. 32905484 PMC7471341

[51] Amir I, Konikoff FM, Oppenheim M, Gophna U, Half EE. Gastric microbiota is altered in oesophagitis and Barrett’s oesophagus and further modified by proton pump inhibitors. Environ Microbiol. 2014; 16:2905–14. 10.1111/1462-2920.12285 24112768

[52] Yang L, Lu X, Nossa CW, Francois F, Peek RM, Pei Z. Inflammation and intestinal metaplasia of the distal esophagus are associated with alterations in the microbiome. Gastroenterology. 2009; 137:588–97. 10.1053/j.gastro.2009.04.046 19394334 PMC2963147

[53] Blackett KL, Siddhi SS, Cleary S, Steed H, Miller MH, Macfarlane S, Macfarlane GT, Dillon JF. Oesophageal bacterial biofilm changes in gastro-oesophageal reflux disease, Barrett’s and oesophageal carcinoma: association or causality? Aliment Pharmacol Ther. 2013; 37:1084–92. 10.1111/apt.12317 23600758

[54] Cook MB, Coburn SB, Lam JR, Taylor PR, Schneider JL, Corley DA. Cancer incidence and mortality risks in a large US Barrett’s oesophagus cohort. Gut. 2018; 67:418–529. 10.1136/gutjnl-2016-312223 28053055 PMC5827961

[55] Shao D, Vogtmann E, Liu A, Qin J, Chen W, Abnet CC, Wei W. Microbial characterization of esophageal squamous cell carcinoma and gastric cardia adenocarcinoma from a high-risk region of China. Cancer. 2019; 125:3993–4002. 10.1002/cncr.32403 31355925 PMC7285383

[56] Li M, Shao D, Zhou J, Gu J, Qin J, Chen W, Wei W. Signatures within esophageal microbiota with progression of esophageal squamous cell carcinoma. Chin J Cancer Res. 2020; 32:755–67. 10.21147/j.issn.1000-9604.2020.06.09 33446998 PMC7797230

[57] Cheung MK, Yue GGL, Lauw S, Li CSY, Yung MY, Ng SC, Yip HC, Kwan HS, Chiu PWY, Lau CBS. Alterations in gut microbiota of esophageal squamous cell carcinoma patients. J Gastroenterol Hepatol. 2022; 37:1919–27. 10.1111/jgh.15941 35816164

[58] Gillespie MR, Rai V, Agrawal S, Nandipati KC. The Role of Microbiota in the Pathogenesis of Esophageal Adenocarcinoma. Biology (Basel). 2021; 10:697. 10.3390/biology10080697 34439930 PMC8389269

[59] Chen C, Chen L, Lin L, Jin D, Du Y, Lyu J. Research progress on gut microbiota in patients with gastric cancer, esophageal cancer, and small intestine cancer. Appl Microbiol Biotechnol. 2021; 105:4415–25. 10.1007/s00253-021-11358-z 34037843

[60] Kashyap S, Pal S, Chandan G, Saini V, Chakrabarti S, Saini NK, Mittal A, Thakur VK, Saini AK, Saini RV. Understanding the cross-talk between human microbiota and gastrointestinal cancer for developing potential diagnostic and prognostic biomarkers. Semin Cancer Biol. 2022; 86:643–51. 10.1016/j.semcancer.2021.04.020 33971261

[61] de Clercq NC, van den Ende T, Prodan A, Hemke R, Davids M, Pedersen HK, Nielsen HB, Groen AK, de Vos WM, van Laarhoven HWM, Nieuwdorp M. Fecal Microbiota Transplantation from Overweight or Obese Donors in Cachectic Patients with Advanced Gastroesophageal Cancer: A Randomized, Double-blind, Placebo-Controlled, Phase II Study. Clin Cancer Res. 2021; 27:3784–92. 10.1158/1078-0432.CCR-20-4918 33883174

[62] Yamamura K, Izumi D, Kandimalla R, Sonohara F, Baba Y, Yoshida N, Kodera Y, Baba H, Goel A. Intratumoral Fusobacterium Nucleatum Levels Predict Therapeutic Response to Neoadjuvant Chemotherapy in Esophageal Squamous Cell Carcinoma. Clin Cancer Res. 2019; 25:6170–9. 10.1158/1078-0432.CCR-19-0318 31358543 PMC6801075

[63] Li Y, Wei B, Xue X, Li H, Li J. Microbiome changes in esophageal cancer: implications for pathogenesis and prognosis. Cancer Biol Med. 2023; 9:j.issn.2095-3941.2023.0177. [Epub ahead of print]. 10.20892/j.issn.2095-3941.2023.0177 37817487 PMC10884538

[64] Pandey A, Lieu CH, Kim SS. The Local Microbiome in Esophageal Cancer and Treatment Response: A Review of Emerging Data and Future Directions. Cancers (Basel). 2023; 15:3562. 10.3390/cancers15143562 37509225 PMC10377659

[65] Sfanos KS. Intratumoral Bacteria as Mediators of Cancer Immunotherapy Response. Cancer Res. 2023; 83:2985–6. 10.1158/0008-5472.CAN-23-1857 37712178

[66] Das PK, Islam F, Smith RA, Lam AK. Therapeutic Strategies Against Cancer Stem Cells in Esophageal Carcinomas. Front Oncol. 2021; 10:598957. 10.3389/fonc.2020.598957 33665161 PMC7921694

