# Supplementary material for: Causal relationship between gut microbiota and risk of esophageal cancer: evidence from Mendelian randomization study
Source: Aging (Albany NY). 2024 Feb 15;16(4):3596–611. doi: 10.18632/aging.205547 (PMC10929825; doi:10.18632/aging.205547)
Supplement: Supplementary Table 3 [file aging-16-205547-s003.docx]

Supplementary Table 3. Detailed summary of MR results (target gut microbiome on esophageal cancer).

| **Taxa** | **Exposure** | **Outcome** | **Nsnp** | **Methods** | **Beta** | **SE** | **OR (95%CI)** | ***P*-value** | **MR-PRESSO** | **Heterogeneity** | | **Horizontal pleiotrop** | | |
| --- | --- | --- | --- | --- | --- | --- | --- | --- | --- | --- | --- | --- | --- | --- |
|  |  |  |  |  |  |  |  |  |  | **Cochran’s Q** | ***P*-value** | **Egger intercept** | **SE** | ***P*-value** |
| Family | Porphyromonadaceae | Esophageal cancer | 9 | MR-Egger | -1.721 | 1.119 | 0.18 (0.02-1.61) | 0.168 | 0.6 | 6.708 | 0.568 | 0.074 | 0.063 | 0.279 |
|  |  |  |  | Weighted median | -0.566 | 0.300 | 0.57 (0.32-1.02) | 0.059 |  |  |  |  |  |  |
|  |  |  |  | Inverse variance weighted | -0.432 | 0.219 | 0.65 (0.42-0.99) | 0.048 |  |  |  |  |  |  |
|  |  |  |  | Simple mode | -0.640 | 0.477 | 0.53 (0.21-1.34) | 0.216 |  |  |  |  |  |  |
|  |  |  |  | Weighted mode | -0.650 | 0.505 | 0.52 (0.19-1.41) | 0.234 |  |  |  |  |  |  |
| Genus | Candidatus Soleaferrea | Esophageal cancer | 11 | MR-Egger | -0.726 | 0.877 | 0.48 (0.09-2.70) | 0.428 | 0.38 | 10.878 | 0.367 | 0.045 | 0.082 | 0.594 |
|  |  |  |  | Weighted median | -0.294 | 0.166 | 0.75 (0.54-1.03) | 0.076 |  |  |  |  |  |  |
|  |  |  |  | Inverse variance weighted | -0.248 | 0.125 | 0.78 (0.61-0.99) | 0.048 |  |  |  |  |  |  |
|  |  |  |  | Simple mode | -0.346 | 0.239 | 0.71 (0.44-1.13) | 0.178 |  |  |  |  |  |  |
|  |  |  |  | Weighted mode | -0.313 | 0.222 | 0.73 (0.47-1.13) | 0.187 |  |  |  |  |  |  |
| Genus | Catenibacterium | Esophageal cancer | 5 | MR-Egger | 0.398 | 1.251 | 1.49 (0.13-17.32) | 0.771 | 0.78 | 1.795 | 0.773 | -0.016 | 0.164 | 0.924 |
|  |  |  |  | Weighted median | 0.270 | 0.168 | 1.31 (0.94- 1.82) | 0.107 |  |  |  |  |  |  |
|  |  |  |  | Inverse variance weighted | 0.196 | 0.134 | 1.31 (1.01- 1.71) | 0.044 |  |  |  |  |  |  |
|  |  |  |  | Simple mode | -0.036 | 0.256 | 1.22 (0.74- 2.01) | 0.487 |  |  |  |  |  |  |
|  |  |  |  | Weighted mode | 0.322 | 0.239 | 1.38 (0.86- 2.21) | 0.249 |  |  |  |  |  |  |
| Genus | Eubacterium coprostanoligenes | Esophageal cancer | 13 | MR-Egger | 0.093 | 0.730 | 1.10 (0.26-4.59) | 0.900 | 0.68 | 9.402 | 0.668 | 0.016 | 0.045 | 0.718 |
|  |  |  |  | Weighted median | 0.474 | 0.231 | 1.61 (1.02-2.53) | 0.040 |  |  |  |  |  |  |
|  |  |  |  | Inverse variance weighted | 0.355 | 0.171 | 1.43 (1.02-2.00) | 0.038 |  |  |  |  |  |  |
|  |  |  |  | Simple mode | 0.675 | 0.425 | 1.97 (0.85-4.53) | 0.138 |  |  |  |  |  |  |
|  |  |  |  | Weighted mode | 0.653 | 0.355 | 1.92 (0.96-3.86) | 0.091 |  |  |  |  |  |  |
| Genus | Marvinbryantia | Esophageal cancer | 10 | MR-Egger | 0.521 | 0.762 | 1.68 (0.38-7.51) | 0.513 | 0.97 | 3.315 | 0.950 | -0.015 | 0.061 | 0.813 |
|  |  |  |  | Weighted median | 0.391 | 0.221 | 1.48 (0.96-2.29) | 0.077 |  |  |  |  |  |  |
|  |  |  |  | Inverse variance weighted | 0.340 | 0.173 | 1.41 (1.01-1.97) | 0.049 |  |  |  |  |  |  |
|  |  |  |  | Simple mode | 0.443 | 0.320 | 1.56 (0.83-2.92) | 0.200 |  |  |  |  |  |  |
|  |  |  |  | Weighted mode | 0.370 | 0.289 | 1.45 (0.82-2.56) | 0.232 |  |  |  |  |  |  |
| Genus | Ruminococcaceae UCG010 | Esophageal cancer | 6 | MR-Egger | 0.632 | 0.935 | 1.88 (0.30-11.77) | 0.535 | 0.69 | 2.971 | 0.704 | -0.010 | 0.059 | 0.863 |
|  |  |  |  | Weighted median | 0.436 | 0.286 | 1.55 (0.88- 2.71) | 0.127 |  |  |  |  |  |  |
|  |  |  |  | Inverse variance weighted | 0.466 | 0.220 | 1.59 (1.03- 2.46) | 0.034 |  |  |  |  |  |  |
|  |  |  |  | Simple mode | 0.347 | 0.424 | 1.42 (0.62- 3.25) | 0.449 |  |  |  |  |  |  |
|  |  |  |  | Weighted mode | 0.304 | 0.404 | 1.36 (0.61- 2.99) | 0.485 |  |  |  |  |  |  |
| Genus | Ruminococcus1 | Esophageal cancer | 10 | MR-Egger | -0.524 | 0.508 | 0.59 (0.22-1.60) | 0.332 | 0.62 | 7.057 | 0.631 | 0.071 | 0.036 | 0.090 |
|  |  |  |  | Weighted median | 0.306 | 0.269 | 1.36 (0.80-2.30 | 0.255 |  |  |  |  |  |  |
|  |  |  |  | Inverse variance weighted | 0.381 | 0.192 | 1.46 (1.00-2.13) | 0.047 |  |  |  |  |  |  |
|  |  |  |  | Simple mode | 0.847 | 0.469 | 2.33 (0.93-5.85) | 0.104 |  |  |  |  |  |  |
|  |  |  |  | Weighted mode | -0.115 | 0.418 | 0.89 (0.39-2.02 | 0.789 |  |  |  |  |  |  |
| Genus | Sutterella | Esophageal cancer | 12 | MR-Egger | -1.233 | 0.972 | 0.29 (0.04-1.96) | 0.233 | 0.93 | 6.918 | 0.805 | 0.108 | 0.063 | 0.116 |
|  |  |  |  | Weighted median | 0.431 | 0.223 | 1.54 (0.99-2.39) | 0.054 |  |  |  |  |  |  |
|  |  |  |  | Inverse variance weighted | 0.414 | 0.166 | 1.51 (1.09-2.10) | 0.012 |  |  |  |  |  |  |
|  |  |  |  | Simple mode | 0.158 | 0.371 | 1.17 (0.57-2.43) | 0.677 |  |  |  |  |  |  |
|  |  |  |  | Weighted mode | 0.183 | 0.329 | 1.20 (0.63-2.29) | 0.589 |  |  |  |  |  |  |
