# Supplementary material for: Causal relationship between gut microbiota and risk of esophageal cancer: evidence from Mendelian randomization study
Source: Aging (Albany NY). 2024 Feb 15;16(4):3596–611. doi: 10.18632/aging.205547 (PMC10929825; doi:10.18632/aging.205547)
Supplement: Supplementary Table 4 [file aging-16-205547-s004.docx]

**Supplementary Table 4. Detailed summary of reverse MR results (esophageal cancer on target gut microbiome).**

| **Exposure** | **Taxa** | **Outcome** | **Nsnp** | **Methods** | **Beta** | **SE** | **OR (95%CI)** | ***P*-value** | **MR-PRESSO** | **Heterogeneity** | | **Horizontal pleiotrop** | | |
| --- | --- | --- | --- | --- | --- | --- | --- | --- | --- | --- | --- | --- | --- | --- |
|  |  |  |  |  |  |  |  |  |  | **Cochran’s Q** | ***P*-value** | **Egger intercept** | **SE** | ***P*-value** |
| Esophageal cancer | Class | Negativicutes | 18 | MR-Egger | -0.048 | 0.039 | 0.95 (0.88-1.03) | 0.236 | 0.604 | 15.619 | 0.550 | 0.004 | 0.009 | 0.611 |
|  |  |  |  | Weighted median | -0.019 | 0.020 | 0.98 (0.94-1.02) | 0.338 |  |  |  |  |  |  |
|  |  |  |  | Inverse variance weighted | -0.029 | 0.014 | 0.97 (0.94-0.99) | 0.048 |  |  |  |  |  |  |
|  |  |  |  | Simple mode | -0.015 | 0.036 | 0.98 (0.92-1.06) | 0.676 |  |  |  |  |  |  |
|  |  |  |  | Weighted mode | -0.012 | 0.032 | 0.99 (0.93-1.05) | 0.694 |  |  |  |  |  |  |
| Esophageal cancer | Order | Selenomonadales | 18 | MR-Egger | -0.048 | 0.039 | 0.95 (0.88-1.03) | 0.236 | 0.566 | 15.619 | 0.550 | 0.004 | 0.009 | 0.611 |
|  |  |  |  | Weighted median | -0.019 | 0.020 | 0.98 (0.94-1.02) | 0.347 |  |  |  |  |  |  |
|  |  |  |  | Inverse variance weighted | -0.029 | 0.014 | 0.97 (0.94-0.99) | 0.048 |  |  |  |  |  |  |
|  |  |  |  | Simple mode | -0.015 | 0.036 | 0.98 (0.92-1.06) | 0.677 |  |  |  |  |  |  |
|  |  |  |  | Weighted mode | -0.012 | 0.031 | 0.99 (0.93-1.05) | 0.677 |  |  |  |  |  |  |
| Esophageal cancer | Genus | Butyricicoccus | 18 | MR-Egger | -0.011 | 0.040 | 0.99 (0.91-1.07) | 0.780 | 0.688 | 14.124 | 0.658 | -0.007 | 0.009 | 0.464 |
|  |  |  |  | Weighted median | -0.028 | 0.021 | 0.97 (0.93-1.01) | 0.177 |  |  |  |  |  |  |
|  |  |  |  | Inverse variance weighted | -0.039 | 0.015 | 0.96 (0.93-0.99) | 0.010 |  |  |  |  |  |  |
|  |  |  |  | Simple mode | -0.057 | 0.037 | 0.94 (0.88-1.02) | 0.139 |  |  |  |  |  |  |
|  |  |  |  | Weighted mode | -0.052 | 0.032 | 0.95 (0.89-1.01) | 0.127 |  |  |  |  |  |  |
| Esophageal cancer | Genus | Eggerthella | 16 | MR-Egger | -0.168 | 0.075 | 0.85 (0.73-0.98) | 0.043 | 0.30 | 17.484 | 0.290 | 0.027 | 0.018 | 0.162 |
|  |  |  |  | Weighted median | -0.076 | 0.040 | 0.93 (0.86-1.00) | 0.058 |  |  |  |  |  |  |
|  |  |  |  | Inverse variance weighted | -0.064 | 0.029 | 0.94 (0.88-0.99) | 0.029 |  |  |  |  |  |  |
|  |  |  |  | Simple mode | -0.073 | 0.071 | 0.93 (0.81-1.07) | 0.320 |  |  |  |  |  |  |
|  |  |  |  | Weighted mode | -0.106 | 0.058 | 0.90 (0.80-1.01) | 0.088 |  |  |  |  |  |  |
| Esophageal cancer | Genus | Eubacterium Xylanophilum Group | 17 | MR-Egger | -0.023 | 0.046 | 0.98 (0.89-1.07) | 0.622 | 0.892 | 9.596 | 0.886 | -0.005 | 0.011 | 0.599 |
|  |  |  |  | Weighted median | -0.038 | 0.023 | 0.96 (0.92-1.01 | 0.112 |  |  |  |  |  |  |
|  |  |  |  | Inverse variance weighted | -0.046 | 0.017 | 0.95 (0.92-0.99) | 0.008 |  |  |  |  |  |  |
|  |  |  |  | Simple mode | -0.043 | 0.039 | 0.96 (0.89-1.03 | 0.283 |  |  |  |  |  |  |
|  |  |  |  | Weighted mode | -0.045 | 0.034 | 0.96 (0.89-1.02) | 0.207 |  |  |  |  |  |  |
| Esophageal cancer | Genus | Intestinimonas | 18 | MR-Egger | -0.010 | 0.047 | 0.99 (0.90-1.09) | 0.822 | 0.56 | 15.844 | 0.534 | 0.0130 | 0.011 | 0.270 |
|  |  |  |  | Weighted median | 0.043 | 0.026 | 1.04 (0.99-1.10) | 0.095 |  |  |  |  |  |  |
|  |  |  |  | Inverse variance weighted | 0.038 | 0.018 | 1.04 (1.01-1.08) | 0.032 |  |  |  |  |  |  |
|  |  |  |  | Simple mode | 0.033 | 0.045 | 1.03 (0.95-1.13) | 0.470 |  |  |  |  |  |  |
|  |  |  |  | Weighted mode | 0.031 | 0.043 | 1.03 (0.95-1.12) | 0.471 |  |  |  |  |  |  |
| Esophageal cancer | Genus | Ruminococcaceae UCG003 | 18 | MR-Egger | 0.004 | 0.043 | 1.00 (0.92-1.10) | 0.914 | 0.628 | 15.184 | 0.582 | 0.007 | 0.010 | 0.481 |
|  |  |  |  | Weighted median | 0.022 | 0.025 | 1.02 (0.97-1.07) | 0.361 |  |  |  |  |  |  |
|  |  |  |  | Inverse variance weighted | 0.034 | 0.016 | 1.03 (1.01-1.07) | 0.042 |  |  |  |  |  |  |
|  |  |  |  | Simple mode | -0.021 | 0.045 | 0.98 (0.89-1.07) | 0.648 |  |  |  |  |  |  |
|  |  |  |  | Weighted mode | -0.012 | 0.038 | 0.99 (0.92-1.07) | 0.753 |  |  |  |  |  |  |
